# Intestinal digestion in poultry compared to other animal species with a diverse diet

**DOI:** 10.3389/fphys.2024.1358524

**Published:** 2024-02-21

**Authors:** Irina V. Kuzmina, Svetlana M. Tolpygo, Alexander V. Kotov, Batogab B. Shoibonov, Tatyana S. Zamolodchikova, Natalya V. Ovchinnikova

**Affiliations:** Laboratory Physiology of Motivations, Federal State Budget Scientific Institution “P. K. Anokhin Research Institute of Normal Physiology”, Moscow, Russia

**Keywords:** poultry, intestine, digestive enzyme activity, mammals, trypsin

## Introduction

The intestines of farm animals and poultry play an important role not only in the assimilation of nutrients from feed but also in maintaining the immune defense of the organism (Grozina, 2014; Fisinin et al., 2016). The barrier function of intestinal villi (microvillus cylindrical epithelium) cannot completely protect the organism from the introduction of pathogenic bacteria and viruses without populating with a variety of beneficial microflora. It is proved that the intestinal microbiota of birds performs numerous functions to maintain homeostasis and resistance. It is known that it takes part in the normal functioning of cardiovascular, endocrine, hematopoietic, nervous, and other systems (Kochish et al., 2020). At the same time, in order to obtain the healthiest birds possible, it is necessary to know their genetic potential and improve nutrition based on physiological features. It is proved that the growth and development of poultry is determined by the formation of the digestive system. At the same time, for the last few decades (30–40 years), serious shifts in the world indicators of productivity of farm animals have been noted (Vertiprakhov et al., 2016a). In order to move forward, it is necessary to clearly represent the physiological digestive processes occurring in the body of any animal, including birds. In this regard, a clear understanding of the digestive processes in the intestine of different animal species, their development in the process of evolution, and the peculiarities of their differences is required (Batoev, 2001). Therefore, it can be considered that the enzymatic activity in the intestine and blood of birds and mammalian animals can be used to “predict” the further growth and development of the organism as a whole. For a deep understanding of the fundamental regularities of food assimilation, it is necessary to know the peculiarities of functioning of the digestive system in animals with different specialization in nutrition.

## Opinion

### Activity of digestive enzymes in the intestine and blood in poultry

The results of studies on fistulated poultry showed that the chyme of the duodenum of meat chickens is characterized by high activity of digestive enzymes, which is consistent with the data from Fisinin et al. (2019). The results obtained on the activity of digestive enzymes in parental lines of broilers (Fisinin et al., 2017a) indicate that hybrids (chickens) have a higher amylase activity in duodenal chyme than hens of maternal and paternal lines. This is most likely due to the diet of poultry (Fisinin et al., 2017b) since the diet of broilers is lower in fiber content and higher in digestible carbohydrates compared to that of the original lines. Since the diet of broiler chickens has a higher crude fat content compared to parental individuals, the lipase activity in the duodenal chyme of hybrids is correspondingly higher as well. A different pattern was observed for the activity of proteolytic enzymes in the intestine. At high crude protein content in broiler diets, protease activity is lower than that in hens of the original maternal line and higher than that in chickens of the paternal line. This may be due to the uneven hydrolysis of different feed components along the digestive tract, where fats are primarily broken down (mainly in the 12-intestine), while amylase and proteases are predominant in the jejunum (Wiseman, 2006). Consequently, the digestive system in meat chickens develops adequately to the nutritional content of incoming feed depending on genetic features that determine the intensity of metabolic processes. The results of studies of amylolytic enzyme activity in the blood plasma in meat chickens have an opposite tendency compared to that in the intestine. In the blood plasma of broiler chickens, the activity of amylase is reduced relative to the indicators in young chickens of maternal and paternal lines. Lipase and protease activities in the plasma had non-significant differences. These results are consistent with the hypothesis of the enteropancreatic circulation of digestive enzymes. The observation of the presence of pancreatic enzymes in the blood confirms the finding that they can be absorbed into the bloodstream, enter the pancreas through the blood, and be secreted into the intestine repeatedly without being broken down to amino acids in the intestine (Esmaeilipour et al., 2012; Vertiprakhov et al., 2016b).

### Activity of digestive enzymes in the intestine and blood in animals with different types of nutrition

In experiments on rabbits, noticeable changes in the activity of blood enzymes at the level of the gastrointestinal tract were revealed. It was determined that blood passing through the capillary network of the digestive tract in rabbits is additionally saturated with protein, but the activity of amylase decreases. The biochemical analysis of the blood of minipigs showed that when blood passes through the gastrointestinal tract, the activity of blood enzymes changes, but the pattern is somewhat different, where the activity of amylase in the proximal small intestine increases. Analyzing these results, we observe that these changes in the activity of enzymes occur not only due to the absorption of nutrients but also as a result of plasma protein biosynthesis, activation, or deactivation of enzymes. The distinctive features of the dynamics of enzyme activity in the outflow blood in rabbits and minipigs are probably related to species-specific morphophysiological features of the digestive tract and differences in animal diets (Ksenofontov and Ksenofontova, 2022). The complex mechanisms of digestion in cattle can be well demonstrated by the dynamics of the activity of digestive enzymes in chyme. Experimental data indicate that the highest activity of protease and lipase is established in the first hours of intestinal chyme sampling, followed by its decrease after 3 h. Amylase activity, on the contrary, decreased in the first 3 h of sampling and increased further 24 h after chyme sampling in the experimental animal (Lebedev et al., 2018).

In numerous experiments on mono- and polygastric animals with duodenal anastomoses implanted behind the ducts of bile and pancreatic juice, it was shown that the size of fluctuations in the content of substances in duodenal chyme is limited. Thus, for 1 kg of dry matter of the consumed feed by cattle, sheep, pigs, and horses, 14 + 0.5 kg of digestive juices is secreted, the amount of which directly depends on the body weight of both mono- and polygastric animals, which leads to the formation of chyme in the duodenum in the amount directly proportional to the body weight of the animal. At the same time, the withdrawal of duodenal chyme leads to digestive disorders; changes in chyme evacuation; disturbance in the cardiovascular system, nervous system, and other pathological phenomena; and later even to lethal outcomes. If the intestine of such animals is being injected with their chyme or chyme taken from another animal, the physiological state normalizes. In addition, it was shown that the nervous system plays a huge role in the regulation of digestive and metabolic functions of the gastrointestinal canal and also revealed the ability of digestive glands to adapt to the food substrate, subtly responding to changes in the chemistry of the diet (Aliev, 2007).

For comparison, an experiment was conducted to keep rats for 10 days on a meat and fish paste diet with 20% higher protein levels used in growing animals. The rats showed a significant increase in pepsin activity in the gastric mucosa and pancreatic homogenate and a decrease in the amylase activity in the small intestinal mucosa. At the same time, studies on mink, when feeding predators a diet with different levels of protein, fat, and carbohydrates, showed that the animals do not show a definite regularity in the changes in the activity of digestive enzymes depending on the ratio of the above components of the diet (Oleinik, 1997). It can be concluded that adaptation of the digestive enzyme spectrum during diet change in mammals requires a long time compared to that in birds. In farm animals (cows, horses, pigs, etc.), adaptation of digestive enzymes occurs much faster, almost as in hens or broilers, with one difference in the level of enzyme activity.

### Trypsin activity in the blood in different types of animals by the type of nutrition

Trypsin is a PAR-activating protease that plays the role of a major digestive enzyme in the duodenum. The results of studies have shown that serum trypsin activity varies between animals. Porcine trypsin contains 4 histidine residues; bovine, sheep, human, and turkey trypsin contain 3 residues; human and turkey trypsin contain 8 polycystin residues; and bovine, pig, and sheep each contain 12 residues (Sukhanova et al., 2018). The results showed that in terms of trypsin activity, the maximum activity in the serum was observed in broiler chickens, which exceeds the level of laying hens by 385.4% in cows, 89.4% in goats, 22.6 in laying hens, and 70% in rats. Biochemical studies of the rat blood serum revealed multidirectional changes in trypsin activity under conditions of thirst and starvation. Thus, the highest activity of this enzyme was observed in animals under water deprivation; on account of food deprivation, on the contrary, a significant decrease in the activity of this enzyme was found in rats. One hour after providing deprived animals with water and feed, a unidirectional significant decrease in enzyme activity was detected in both experimental groups (Kuzmina and Ovchinnikova, 2023). The dominant position of birds in the activity of this enzyme indicates a higher level of metabolism in relation to mammalian animals. It has been established that the indicator of trypsin activity in the blood can be used to judge the state of intestinal health in birds and the processes of digestive adaptation to the composition of the diet (Trukhachev et al., 1999; Vertiprakhov et al., 2023).

## Conclusion

The biological law that determines the development of the organism depending on the genotype and environmental factors provides birds with more intensive digestive processes in the metabolic system compared to other animal species. Since digestion of feed nutrients depends on the intensity of hydrolysis in the intestine, digestive processes in birds are 10 times faster compared to horses and ruminants and 2–3 times more active than digestion in pigs and rats.
